# Supplementary material for: Patterns of Intron Gain and Loss in Fungi
Source: PLoS Biol. 2004 Nov 30;2(12):e422. doi: 10.1371/journal.pbio.0020422 (PMC532390; doi:10.1371/journal.pbio.0020422)
Supplement: Table S1 — Also available at http://genes.mit.edu/NielsenEtAl/. (4.3 MB ZIP). [file pbio.0020422.st001.zip › NielsenEtAl/html/15.html]

AN6177.1.NCU05899.1.MG00835.1.FG01705.1


```
 CLUSTAL W (1.82) Multiple Sequence Alignments - Introns Inserted


Sequence 1: MG00835.1	498 aa
Sequence 2: FG01705.1	526 aa
Sequence 3: NCU05899.1	525 aa
Sequence 4: AN6177.1	423 aa
Alignment Length: 565 aa
Number Identitical Residues: 174 aa
Alignment Score (without introns) 9708


MG00835.1 	-MS~YRVAAPDEYLAITGMGVKTI~RITKASWVLPFQR~CMRFS~VQPHDYAMNLQAMTR
NCU05899.1	MAS~YKIAAPDEYLAITGMGVKTL1KITKAAWVWPFQR~CMRFS~VQPHDYAMSLQAMTK
FG01705.1 	-MS~YRISAPDEYLAITGMGIKTV~KITKAAWVWPLQR~CTRFS2IRPHDYAMDLQAMTK
AN6177.1  	-MR2YCIAEPSEYLVLTGAGIADI~EICKKAWVWPWQR0CARIS~VMPFDFSLSLQAMTI
          	    * :: *.***.:** *:  : .* * :** * ** * *:* : *.*:::.***** 

MG00835.1 	EKLLFQLPVVFTIGPDINQRGANS---------KGQPVHEPENEGDEEAR-EDRGDALMK
NCU05899.1	EKLQFLLPVVFTVGPDVNQRGANIRMFHDEPAKRSNEDDVATAVSDSAVRREDRGDALMK
FG01705.1 	EKLQFSLPVVFTVGPDINQRGANQ---------RGG----PDDEADDLVR-EDRGDALMK
AN6177.1  	EKLQFSLPAVFTIGPEDKLSSLKK--------------------YALLLSGKTGHQPSTR
          	*** * **.***:**: :  . :                            :   :.  :

MG00835.1 	YAMLLADGANEGKKSSGSQQFLENIVKGIIEGETRVLVSSMTMEEIFTEREVFKRRIFKN
NCU05899.1	FAMLLAD--SGRDKGPNNHDFLEGIVKGIIEGEVRVLVSAMTMEEIFSEREVFKRRIFRN
FG01705.1 	YAMLLTS--SEDKEHSSQSQHVANIVKGIIEGETRVLVSSMTMEEIFTEREVFKKRIFRN
AN6177.1  	PAAATED-----LNTPTTRNHVQDIVKGIIEGETRVIVSSMTMEEIFKERQIFKSKVISN
          	 *    .      : .   :.: .*********.**:**:*******.**::** ::: *

MG00835.1 	IRGELAQFGLKI~YNSN~-----VKELKDAPNSSYFESLSRKAHEGATNQARIDVAEAQL
NCU05899.1	IQSELDQFGLKI~YNAN~-----VKELKDAPGSTYFASLSQKAHEGATNQARIDVAEAQL
FG01705.1 	IQNELSQFGLLI~FNSN0LTVPQVKELKDAPDSVYFASLSRKAHEGATNQARIDVAEAQL
AN6177.1  	VQNELEQFGLKI2YNAN~-----VKELQDTKGSEYFAFLSRKAQEGALNQAKIDVAEARM
          	::.** **** * :*:*      ****:*: .* **  **:**:*** ***:******::

MG00835.1 	RGNVGEAQRKGE-----------EQREIAKINAETAVQKVDRDIERAQAESNLATRKTLL
NCU05899.1	RGNVGTQKRKGEEAREVAKIQGEQDRELAKIQAETQVQKTERDIEKATAEALLKTRQVEL
FG01705.1 	RGNVGEAQRKGE-----------QEREIAKINAETAVQKTERDIEKAQAESKLDTKRTGL
AN6177.1  	RGEIGEAEKKGR-----------TKQEISKIDAETAVLETKRKAEKAKADSELLNRQTEL
          	**::*  ::**.            .:*::**:*** * :..*. *:* *:: * .::. *

MG00835.1 	NRDVEIARIEATRKTEAKDEDLKKEVQVKKAAAEMERLRATDVVKATIERESAQQSADAK
NCU05899.1	DRDVQIAGIQAARNTEAEDETLKREVQIKRAAAEMERLRATDVVKASIAREAKQQAADAK
FG01705.1 	TRDVDLARIQAQRSLESQDEDLKRDVEKKRAAAEMERLRATDVVRATIERESKQQAADAS
AN6177.1  	DSQVEMGKIIAKRQTESRDAELQKQVETKRAETELERLRAKQVTKSKVERESAQEQADAL
          	  :*::. * * *. *:.*  *:::*: *:* :*:*****.:*.::.: **: *: *** 

MG00835.1 	AYAVQVEARADYDR-------------------------------------TRLDADAES
NCU05899.1	AYEIEKEAQANYEKAK--------------------------QHTEADVYETKAGADAQA
FG01705.1 	AYEIEADARARQEASQRKADAAAYQTKISAEADATASYAKVTKNTDAAAYQTRNDAEAYN
AN6177.1  	YYSNTKAAEG-------------------------------------RLYEEKMEADARF
          	 *     *..                                        . :  *:*  

MG00835.1 	~YALVKRAEAELTRRTKEAEAMMAMAEAYGQMSKAFGGPQGLLAYLMIEKGTYVELAKAN
NCU05899.1	~YAAIRLADAELQQKLRAAEGMSAMAEAYAKMSHAFGGPQGLLQYMMIEKGTYVELAKAN
FG01705.1 	~YAAQQRAEAQLVAKLREAEGIAAMAEAYGKLSNAFGGPAGLLQYMMIEKGTYVELAKAN
AN6177.1  	1YRSCKDDDAMFNTKKREAEGVMEMAKAYGALIDVLGGPDAFLQWKMIETGMYERLALAN
          	 *   :  :* :  : : **.:  **:**. : ..:*** .:* : ***.* * .** **

MG00835.1 	AEAVQGLQPKISVWNTG---AAEGGSSQGQGGIEAMRNVYQMLPPLMTTINEQTGITLPE
NCU05899.1	AEAIRGLQPKISVWNTGAEAGSSGGAGEQQSSMATMRNIYQMLPPLMTTINEQTGITLPE
FG01705.1 	ASAIRGLEPKISVWNTG---SAQGG--QGADATETMRNVYQMLPPLMSTINDQTGITLPE
AN6177.1  	GQAIQGLQPKITTWNTG------------------------GKWPRLFTIGPH-------
          	..*::**:***:.****                           * : **. :       

MG00835.1 	WQFGRMNNDMAAVEREKAIASNSKRHAESNGA
NCU05899.1	WQFGRMAGQMSEVERRGQASNGQKE-------
FG01705.1 	WQFGKMSAGVNAVGQEGAKVNGHKGQ------
AN6177.1  	--------------------------------
          	
```
